# Nanoplastics and Neurodegeneration: A Roadmap From Mechanism to Causation

**DOI:** 10.1002/advs.75151

**Published:** 2026-04-09

**Authors:** Yuhuan Li, Yue Wang, Xiufang Liang, Mengze Xu, David T. Leong, Pu Chun Ke

**Affiliations:** ^1^ Liver Cancer Institute, Key Laboratory of Carcinogenesis and Cancer Invasion, Zhongshan Hospital, Ministry of Education Fudan University Shanghai China; ^2^ Drug Delivery, Disposition and Dynamics, Monash Institute of Pharmaceutical Sciences Monash University Parkville Victoria Australia; ^3^ School of Biomedical Sciences and Engineering, Guangzhou International Campus South China University of Technology Guangzhou China; ^4^ Department of Biology, Faculty of Arts and Sciences Beijing Normal University Zhuhai China; ^5^ Department of Chemical and Biomolecular Engineering National University of Singapore Singapore Singapore

**Keywords:** ageing, Alzheimer's disease, causation, environmental health, nanoplastic, nanotoxicology, neurodegeneration, Parkinson's disease

## Abstract

Nanoplastics are ubiquitous by‐products of global plastic production and have emerged as a potentially consequential yet insufficiently defined threat to health. Recent studies have revealed that these synthetic particulates can cross the blood‐brain barrier, accelerate amyloid aggregation, impair microglial clearance, hijack the gut‐liver‐brain axis, and drive neuroinflammation—mechanisms central to neurodegeneration in Alzheimer's and Parkinson's disease. In addition, anionic nanoplastics can induce vascular endothelial leakiness, thereby harboring a paracellular route for their systemic and cerebral access. Yet causality remains unproven in implicating nanoplastics for neurodegeneration in the absence of standardized human exposure data, mechanistic specificity, and epidemiological evidence, especially considering the supra‐environmental doses employed. Here, we synthesize current knowledge, examine barriers to causal understanding, and propose a roadmap to advance this emerging scientific frontier of great public concern and inform future strategies for sustainable materials innovation.

## Graphical abstract

Nanoplastics are pervasive environmental contaminants with potentially profound implications for human health. Emerging evidence suggests a possible link between nanoplastic exposure and neurodegeneration, a key driver of ageing and dementia, yet causality remains unresolved. This Perspective synthesizes current evidence, identifies critical knowledge gaps and deficiencies, and outlines a multidisciplinary roadmap to clarify causal links and guide safer materials innovation.

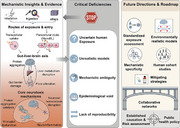

## 1 Introduction

Alzheimer's disease (AD) and Parkinson's disease (PD) are the two most prevalent neurodegenerative disorders associated with dementia, yet the mechanisms underlying their pathogenesis remain incompletely understood [1, 2]. While amyloid‐β (Aβ) and α‐synuclein (αS) aggregation, tauopathy, neuroinflammation, and genetic predisposition are recognized as primary drivers, accumulating evidence indicates that environmental exposure to air pollutants, pesticides, and metals also plays a significant role in disease risk [3, 4, 5, 6, 7, 8]. Recent reports of microplastics (>1 µm) and nanoplastics (<1 µm) detected in human blood, placenta, lung, liver, and brain tissue [9, 10, 11] converge on the possibility that chronic plastic exposure represents a previously unrecognized contributor to human diseases, including neurological disorders.

To better define our subject at discussion, it is important to distinguish nanoplastics from plastic‐derived molecular leachates such as monomers, oligomers, plasticizers, stabilizers, and flame retardants [12]. Unlike these molecular leachates, nanoplastics behave like colloidal particles that can attract protein coronas [13] (i.e., layers of spontaneously adsorbed proteins and other amphiphiles on the nanoparticle surface in biological fluids, which subsequently dictate the biological identity of the nanoparticle), undergo endocytic uptake, translocate across biological barriers, and catalyze protein misfolding [14, 15, 16, 17]. In real‐world scenarios, humans are likely exposed to both plastic entities, hence complicating causal attribution [12, 18].

Plausible human exposure pathways to nanoplastics include ingestion of food and drinking water, inhalation of indoor and occupational aerosols, contact with synthetic textiles or recycling streams, and exposure through certain medical or consumer products [18, 19, 20]. Nanoplastics are not expected to be metabolized like small molecules; rather, their fate depends on particle size, surface chemistry, corona evolution, sequestration, clearance, and slow physicochemical transformation [15, 19, 21]. At present, no consensus exists on which real‐world route yields the greatest brain‐relevant internal dose [19, 21, 22].

Intensive research in recent years has shown that nanoplastics can cross biological barriers, impair proteostasis, and amplify neuroinflammatory responses [18, 23, 24, 25, 26, 27]. Nevertheless, the field faces major translational gaps that hinder definitive conclusions beyond empirical observations and hypotheses: human exposure levels remain poorly defined, experimental models often lack environmental relevance, and robust epidemiological data are lacking. Accordingly, this *Perspective* synthesizes mechanistic and toxicological evidence, identifies key deficiencies that hinder causal inference, and outlines a roadmap to determine whether nanoplastics truly act as causal contributors to AD, PD, and other neurological disorders. As the biological effects of nanoplastics—especially their neurotoxicity—have been reviewed elsewhere [28, 29, 30, 31], those aspects will be discussed only when essential.

## 2 Mechanistic Insights Into Nanoplastics‐Induced Neurotoxicity

Current mechanistic insights into nanoplastics‐induced neurotoxicity are informative, but they are largely derived from studies of polystyrene‐based simplified model systems and, therefore, may not capture the full physicochemical complexity of environmentally relevant nanoplastics [15, 32]. Environmental nanoplastics, by contrast, are far more heterogeneous in polymer type, size, shape, weathering state, and surface properties [14, 18, 21, 32]. Accordingly, these diverse features may shape nanoplastic protein corona composition, barrier and interfacial interactions, cellular uptake, and downstream neurotoxicity, among other factors.

### 2.1 Blood‐Brain Barrier (BBB) Translocation

Rodent studies have revealed that orally, intranasally, and parenterally delivered nanoplastics can translocate across the BBB to deposit in hippocampal and striatal regions [33, 34, 35]. BBB disruption, in particular, encompasses oxidative stress, tight‐junction disassembly, and endothelial activation [36, 37], all of which have been linked to nanoplastic exposure in the brain. Recent post‐mortem detection of plastics in the human brain [38], although controversial in the methodology applied, has generated significant public concern and underscores their clinical relevance. Still, current evidence remains limited in the use of heterogeneous nanoplastic sizes, surface chemistries, and exposure paradigms, making it impractical to compare findings across studies and define physiologically relevant exposure thresholds.

### 2.2 Nanoplastics‐Induced Vascular Leakiness

**FIGURE 1:**
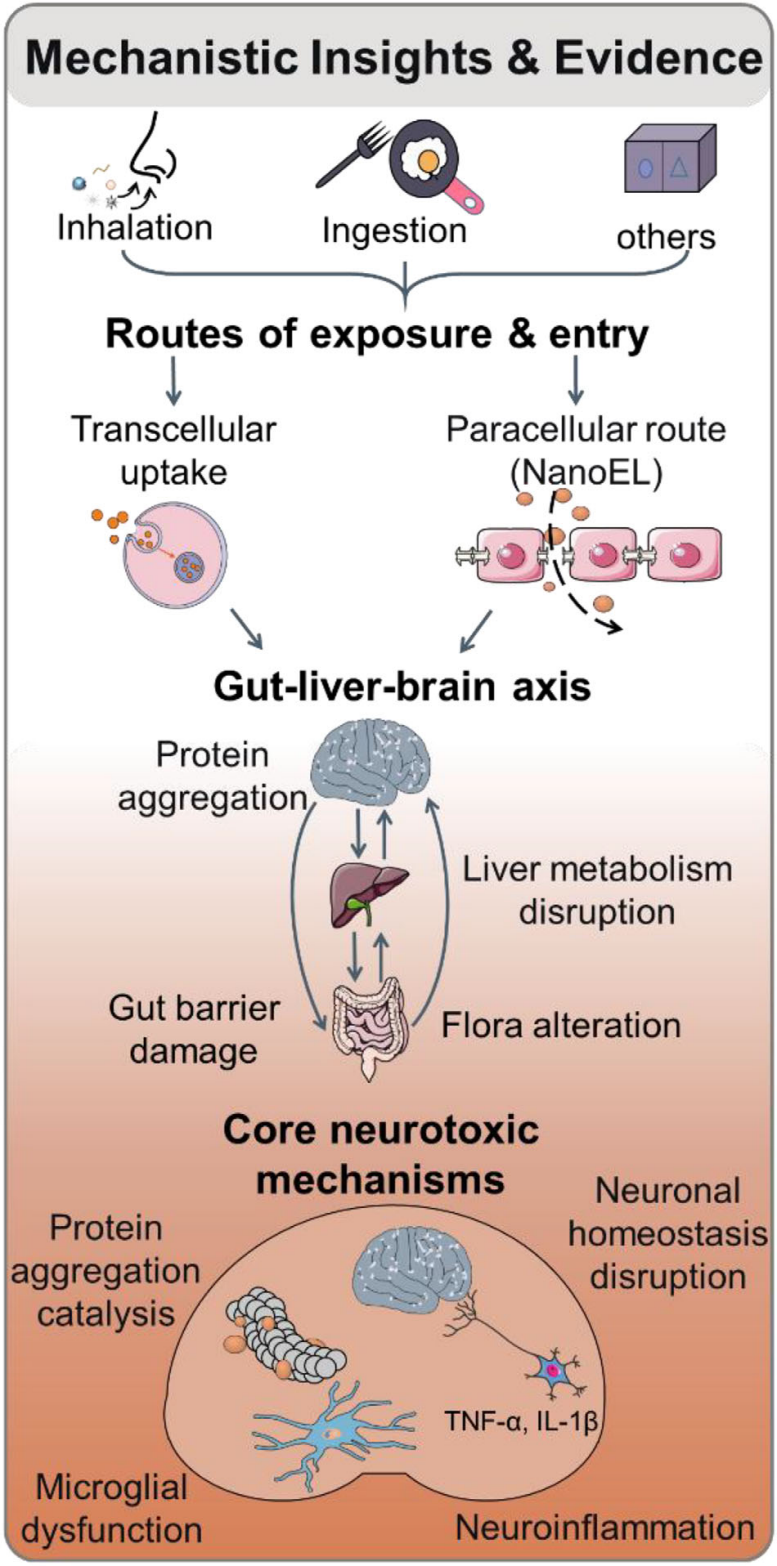
Mechanical insights into the neurological implications of nanoplastic exposure. Emerging evidence increasingly points to mechanisms underlying the pathologies of AD, PD, and other neurological disorders. Nanoplastics may enter the body through inhalation, ingestion, and other exposure routes. Upon entry, these particles may traverse biological barriers via transcellular uptake or paracellular transport, such as NanoEL, thereby facilitating their interactions with peripheral tissues and the gut‐liver‐brain axis. Along this axis, nanoplastic exposure entails gut barrier damage, alterations in intestinal microbiota composition, disruption of hepatic metabolism, and promotion of protein aggregation. These peripheral and systemic disturbances may converge on core neurotoxic mechanisms in the brain, including catalytic enhancement of pathogenic protein aggregation, disruption of neuronal homeostasis, microglial dysfunction, and neuroinflammation accompanied by increased release of pro‐inflammatory mediators. NanoEL: nanomaterial‐induced endothelial leakiness.

Anionic polystyrene and poly(methyl methacrylate) nanoplastics can trigger cerebrovascular leakiness via NanoEL, that is, rupture of paracellular junctions driven by multi‐point interactions between the synthetic particulates and vascular endothelial (VE)‐cadherin proteins (Figure 1) [39, 40, 41]. This process, observed in vitro, ex vivo, in silico, and in vivo, is initiated when nanoplastics smaller than 100 nm in size enter the paracellular endothelial space and interact with the EC1‐2 domains of homophilic VE‐cadherins. By comparison, cationic polystyrene and poly(methyl methacrylate) nanoplastics do not trigger NanoEL, as their membrane affinity promotes direct uptake via endocytic and transcytotic pathways. Interestingly, NanoEL induced by nanoplastics, at least on the timescales of sub to a few hours, is independent of the toxicological hallmarks of oxidative stress, autophagy, or apoptosis [39], reflecting their biophysical‐biochemical nature. Thus, NanoEL represents a novel route of systemic propagation that complements transcellular uptake (Figure 1), enriching our understanding of how environmental nanoplastics may traverse the central nervous system (CNS) to impact cerebrovascular function.

### 2.3 Protein Aggregation Catalysis

Owing to their large surface area and hydrophobicity, nanoplastics can function as catalytic substrates for the adsorption and subsequent misfolding of amyloidogenic proteins (Figure 1). In vitro data reveal that polystyrene nanoplastics can exacerbate the aggregation of Aβ and accelerate αS fibrillization [16, 17, 42], hallmarks of the AD/PD pathology. Exposure to anionic and cationic polystyrene nanoplastics also amplifies protein aggregation in transgenic mouse models, suggesting convergence with core AD/PD pathology [42, 43]. This effect, while distinct from oxidative stress, synergizes with the latter to promote neurodegeneration through the interplay of amyloid aggregation and reactive oxygen species (ROS) production [44, 45].

### 2.4 Disruption to Neuronal Homeostasis

Chronic exposure to nanoplastics perturbs neuronal homeostasis through converging mechanisms that resemble the hallmarks of both AD and PD (Figure 1). Specifically, single‐nucleus transcriptomic analyses reveal that polystyrene nanoplastics disrupt energy metabolism in dopaminergic neurons, particularly oxidative phosphorylation and mitochondrial function [23, 46]. These alterations compromise ATP production and redox balance, thereby sensitizing neurons to metabolic stress. Concurrently, genes regulating synaptic signaling and neuroinflammatory responses are dysregulated, suggesting that mitochondrial dysfunction may act upstream of both synaptic impairment and immune activation [23, 46]. Additional studies reveal that polystyrene nanoplastics can localize in mitochondria, destabilize membrane potential, and promote ROS accumulation, collectively driving PD‐like neurodegeneration [23, 42, 47]. Consistently, exposure to micro‐ and nanosized polyethylene terephthalate impairs mitochondrial functions in human brain vascular pericytes, reducing mitochondrial respiration and decreasing ATP production [48]. Additionally, exposure to polystyrene nanoplastics promotes AD‐relevant pathologies by accelerating Aβ misfolding and aggregation, seeding oligomers and fibrils that compromise synaptic integrity [17]. Single‐cell analyses indicate disruption of proteostasis, autophagy‐lysosomal pathways, and cytoskeletal dynamics, while tau hyperphosphorylation further destabilizes axonal transport. Mitochondrial dysfunction and oxidative stress exacerbate these effects, creating a vicious cycle between metabolic failure, protein aggregation, and chronic neuroinflammation [16, 17]. Together, these findings suggest that nanoplastics can act as multifaceted stressors, linking energy failure, synaptic disruption, protein misfolding, and neuroinflammation across disease contexts.

### 2.5 Microglial Dysfunction and Impaired Clearance

As the principal immune cells of the CNS, microglia mediate host defense by phagocytosing amyloid aggregates and plaques. However, exposure to nanoplastics—including polyethylene, polypropylene, polystyrene, polyvinyl chloride, polylactic acid, and polyethylene terephthalate—disrupts ATP production, inhibits transporter activity, and reduces phagocytosis of microglia, thereby exacerbating neuroinflammation [16, 49]. In human cerebral organoids, polypropylene nanoplastics disrupt glial differentiation and neuroimmune balance, resulting in reduced oligodendrocyte and astrocyte markers and increased allograft inflammatory factor 1 (AIF1) [50]. As a result, dysregulated microglia not only fail to clear pathological proteins but also release cytokines that further impair neuronal health, creating a self‐amplifying toxic cycle (Figure 1).

### 2.6 Systemic Propagation via the Gut‐Liver‐Brain Axis

Gut‐administered nanoplastics induce aggregation of A53T αS (a genetic driver of early‐onset PD) within the enteric nervous system, promoting prion‐like propagation to the brain along Braak's gut‐brain axis [51, 52, 53]. Polystyrene nanoplastics also promote Aβ amyloidosis and neuroinflammation, giving rise to the central to peripheral spread of the AD pathophysiology via gut‐liver‐brain signaling (Figure 1) [43]. In addition, chronic exposure to polyethylene microplastics and nanoplastics alters the diversity of intestinal microbiota [54]. Together with NanoEL, these findings point to multiple systemic routes through which nanoplastics may influence neurodegenerative processes, operating through both top‐down and bottom‐up mechanisms. PD, specifically, is increasingly recognized as both a neurological and metabolic disorder. It is therefore sensible that polystyrene nanoplastics exacerbate PD pathology by disrupting metabolism along the gut‐liver‐brain axis [55]. In an A53T αS mouse model, polystyrene nanoplastics compromised gut barrier integrity, promoted *Desulfovibrio* overgrowth, and altered over 200 types of fecal metabolites. Perturbations in cytochrome P450 activity, bile acid pathways, apoptosis, and lipopolysaccharide biosynthesis linked microbial dysbiosis to systemic metabolic stress. Concurrent liver inflammation further indicated disrupted metabolic homeostasis. These perturbations drove αS aggregation and neuroinflammatory responses, highlighting the potential role of nanoplastics in aggravating PD via both metabolic and neurological dysfunction [55]. Future studies, therefore, should integrate longitudinal in vivo models with multi‐organ and multi‐omics approaches to determine how gut, liver, and brain responses evolve over time and whether these peripheral alterations causally drive central neurotoxicity.

### 2.7 Developmental, Gender, and Transgenerational Effects

Maternal nanoplastic exposure has been reported to disrupt offspring neurodevelopment and behavior in mice [56]. Prenatal exposure to polypropylene nanoplastics inhibited neuronal development and cell proliferation in fetal mouse cerebral cortex, with CYSLTR1 and PTH1R identified as the potential molecular targets mediating the neurotoxic effects. Such early‐life insults led to pronounced functional deficits in the animal offspring, including impaired spatial memory, reduced motor coordination, and enhanced anxiety. While dementia is a gender‐biased disease [57], nanoplastics also entail sex‐specific effects on the brain and neurological function [58]. Specifically, under co‐exposure to 4‐methylbenzylidene camphor and polystyrene nanoplastics, female zebrafish exhibited autism‐spectrum‐like behaviors, whereas male zebrafish displayed PD‐like symptoms [58].

## 3 Deficiencies Obstructing Causation

**FIGURE 2:**
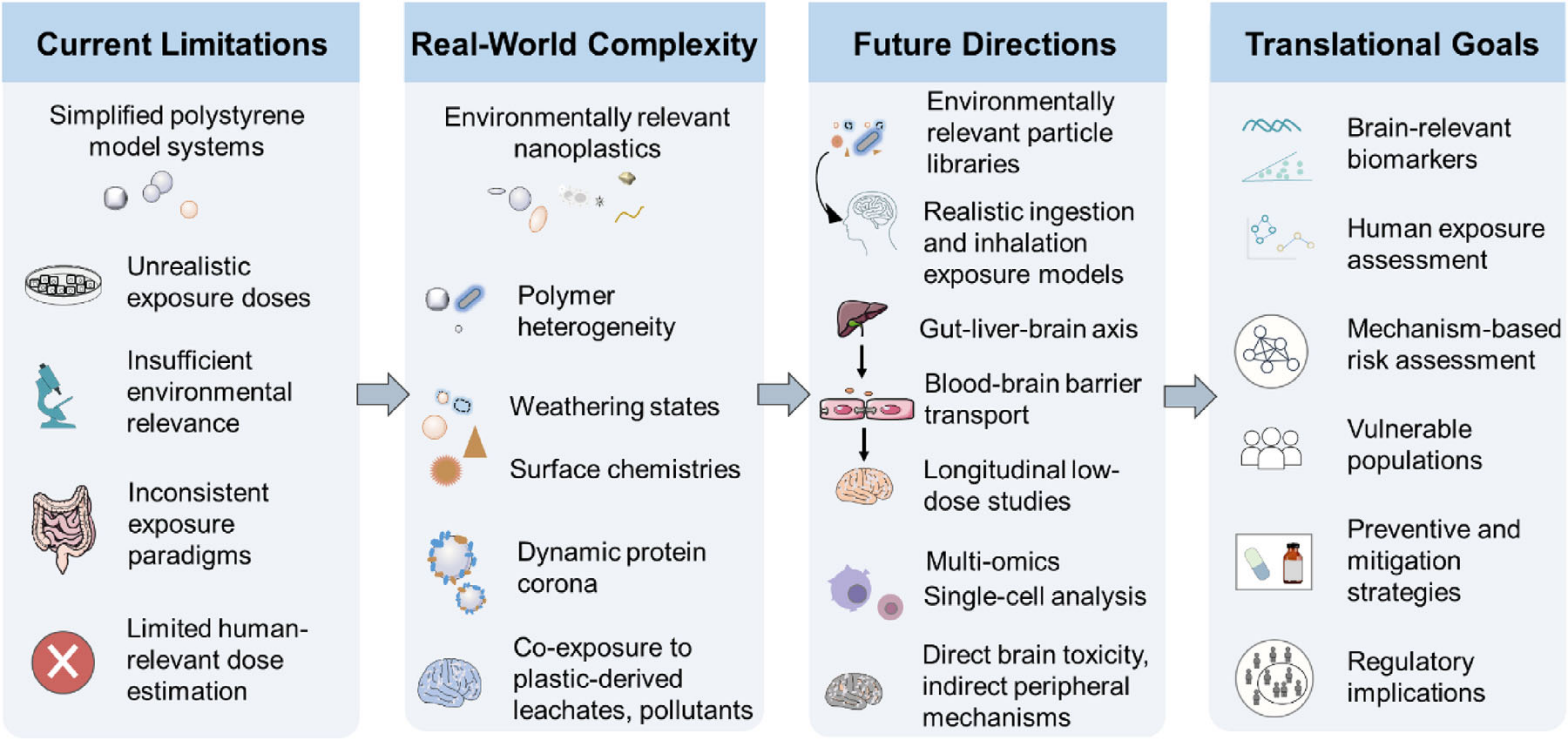
Overview of the central components of this *Perspective*, highlighting key gaps in the causal chain and roadmap for identifying nanoplastics‐induced neurological risks.

Below, we identify the major deficiencies preventing the field from moving beyond mechanistic plausibility to causation (Figure 2), extracted from in vitro, in silico, and in vivo studies.

### 3.1 Uncertain Human Exposure

Although nanoplastics have been detected in animal and human tissues, standardized quantitative biomarkers are lacking, and contamination risks remain high due to the widespread use of plasticware in research laboratories. Differences in detection techniques and analytical platforms also hinder direct comparison across studies. Further, the environmental ubiquity of nanoplastics does not imply known internal doses, tissue burdens, or brain‐relevant exposure.

### 3.2 Unrealistic Experimental Models

As stated earlier, most studies to date have been using polystyrene nanoplastics, overlooking the heterogeneity of environmentally weathered plastic particles. Other polymer types, including polyethylene and polypropylene, may exhibit toxicokinetic profiles distinct from polystyrene resulting from their diverse physicochemical properties and entailed interactions with amyloid proteins, cell membranes, and cellular organelles. Further, distinct polymeric species develop unique cellular protein coronas [13] due to their differences in surface charge, amphiphilicity, and tendency to mediate hydrogen bonding and hydrophobic interactions, potentially yielding divergent pathological outcomes (Figure 2).

### 3.3 Supra‐Physiological Dosing

High‐dose intravenous or intracerebral administration bypasses natural exposure routes and inflates toxicological effects. Indeed, recent studies have demonstrated the importance of environmentally realistic dosing for accurate risk assessment of micro‐ and nanoplastics [59, 60]. This deficiency in experimental design likely arises from a lack of metadata on the environmental abundance of nanoplastics.

### 3.4 Mechanistic Ambiguity

As oxidative stress, inflammation, and proteostasis disruption are common hallmarks shared by many neurological and environmental toxicants, disease‐specific causality has yet to be established for nanoplastics profiled by these parameters. This is compounded by the overlap between AD and PD pathology [61], which further intertwines with metabolic disorders such as type 2 diabetes through systemic pathways and the gut‐brain axis, obscuring causal relationships [62, 63]. Although NanoEL offers a molecular mechanism for vascular permeability, its relevance under conditions of chronic low‐dose human exposure remains to be validated. The key challenge is not to simply compile common injury pathways, but to identify nanoplastic‐specific initiating events and molecular fingerprints that distinguish nanoplastic‐induced neurotoxicity from other exposures, such as PM_2.5_ or heavy metals. Without specificity, mechanistic studies remain descriptive and cannot support causal inference.

### 3.5 Epidemiological Void

There is an epidemiological void regarding the contribution of nanoplastics to neurodegenerative disorders. To date, no cohort studies, case‐control analyses, or population‐level investigations have directly assessed whether human exposure to nanoplastics correlates with disease incidence, lagging considerably behind, for example, research on PM_2.5_ air pollution and its links to Lewy body dementia and PD [64, 65]. Available data on exposure are limited to environmental or occupational measurements and rarely incorporate biomarkers that indicate internalized nanoplastics, particularly in neural tissues. Longitudinal monitoring across life stages is lacking, considering the decades‐long latency of neurodegenerative diseases. Critical confounders, including co‐exposures to other environmental toxicants, genetic susceptibility, and lifestyle factors, remain unquantified, further hindering any inference about disease risk.

### 3.6 Lack of Reproducibility

Reproducibility is hindered by widespread heterogeneity in nanoplastic characterization, dosing regimens, and reporting practices. As noted earlier, studies often differ in particle size, shape, polymer type, surface charge, and functionalization, yet these critical properties are not consistently reported. Exposure doses often vary and exceed environmentally relevant levels, and routes of administration range from oral gavage to injection, further complicating cross‐study comparisons. Additionally, experimental methods for quantifying internalization, toxicity, and molecular effects are inconsistent. This lack of standardization prevents meaningful meta‐analyses from identifying rigorous trends or dose‐response relationships. Without common protocols and comprehensive reporting standards—including full physicochemical characterization, exposure conditions, and methodological details—drawing conclusions about nanoplastic neurotoxicity remains untenable, undermining the reliability and translational value of the field.

## 4 Future Directions

### 4.1 Standardizing Exposure Assessment

Standardizing exposure assessment represents a bottleneck in evaluating the neurodegenerative risk of nanoplastics. Currently, no consistent detection pipelines or certified reference materials exist that reflect the environmental heterogeneity of nanoplastics, making cross‐study comparisons unreliable. Longitudinal exposure mapping in sentinel populations could provide an essential first step toward establishing epidemiological correlations with AD and PD incidence, yet such efforts are lacking. Similarly, animal models rarely employ chronic, low‐dose exposure paradigms or incorporate sufficient longitudinal tracking of behavioral changes, neuropathology, and molecular biomarkers, limiting the ability to establish temporal cause–effect relationships. The absence of standardized reporting for particle characterization, dosing, and experimental endpoints further hampers reproducibility and meta‐analysis. Addressing these deficiencies through consistent detection methods, validated reference materials, and longitudinal experimental designs (Figure 2) would not only improve translational insight from animal and in vitro studies but also lay the groundwork for human studies capable of establishing whether nanoplastics are true risk modifiers for neurodegenerative disease.

### 4.2 Environmentally Realistic Models

Incorporating weathered, multi‐polymeric nanoplastics and chronic low‐dose regimens in animal studies will better represent real‐world conditions and mimic human environmental exposure. Vascular leakiness assays may determine the relevance of NanoEL as a key translocation pathway for nanoplastics to enter the brain. Complementary in vitro platforms, such as organoids and microfluidic BBB models, can provide cost‐effective, high‐throughput alternatives that allow mechanistic dissection of nanoplastic transport and toxicity while reducing animal usage. Together, these approaches are expected to improve experimental relevance, reproducibility, and translational insight, bridging the gap between mechanistic findings and potential human health impacts (Figure 2).

### 4.3 Mechanistic Specificity

Mechanistic specificity in nanoplastic neurotoxicity remains largely undone. The priority is to identify molecular fingerprints that distinguish nanoplastic‐triggered pathology from other neurotoxic exposures. Ultrasensitive protein misfolding assays such as RT‐QuIC and PMCA, combined with multiscale computational simulations, multi‐omics, and in*/*ex vivo imaging, could determine whether nanoplastics seed pathogenic protein aggregation, alter amyloid structural polymorphs, and induce cellular stress granules via liquid‐liquid phase separation (thermodynamically driven partitioning processes that render biomolecular condensates, which then facilitate stress granule assembly or aberrant protein aggregation) [66]. Additional approaches may be employed to interrogate VE‐cadherin disruption in conjunction with ROS‐elicited cerebrovascular damage and further decode the correlation between cerebral amyloid angiopathy and AD/PD pathology using cellular, organoid, and animal models. Genetic or pharmacological blockade of nanoplastic‐endothelial interactions may provide a direct test of causality, linking vascular perturbation to downstream proteinopathy and neurodegeneration. Formation of nanoplastic‐protein coronas and dynamics of nanoplastic‐seeded amyloid aggregation, along with the associated signaling pathways and their effects on CNS homeostasis, should be systematically investigated using computational, cellular, and animal models, complemented by single‐cell and spatial multi‐omics approaches. Matched internal‐dose comparisons with PM_2.5_, metals, and other nanomaterials will be essential to identify nanoplastic‐specific mechanisms. Establishing such fingerprints could move the field beyond descriptive toxicology toward causal inference. Collectively, we anticipate that these strategies could resolve mechanistic ambiguities, differentiate nanoplastic‐specific effects from general oxidative or inflammatory responses, and establish a framework for connecting molecular events to translational outcomes in neurodegenerative research.

### 4.4 Human Cohorts and Biobanks

Human cohort studies and biobanks provide an essential yet largely untapped resource for establishing associations between nanoplastic exposure and neurological disorders. Leveraging longitudinal cohorts with stored biospecimens to assess nanoplastic levels alongside vascular permeability markers could constitute a critical step in establishing a connection between exposure and the development of AD or PD. Integration of multi‐omics approaches including transcriptomics, proteomics, and metabolomics could further reveal molecular signatures associated with nanoplastic exposure, uncover early biomarkers of pathology, and help differentiate nanoplastic‐specific effects from general environmental toxicants (Figure 2).

### 4.5 Mitigating Strategies

Mitigation of nanoplastic‐entailed neurotoxicity should be mechanism‐driven rather than reactive. The molecular processes of protein corona formation, VE‐cadherin disruption, amyloid surface catalysis, and gut‐brain metabolic perturbation may also define actionable design principles for both safer polymer engineering and biomedical intervention.

Mechanistic studies have revealed that surface charge and hydrophobicity dictate nanoplastic corona composition, which in turn governs their biodistribution, endothelial interaction, and amyloid seeding. If pathological outcomes are to be circumvented at the bio‐nano interface, then safer‐by‐design plastics must minimize high‐risk corona signatures. Rational strategies include reducing nanoplastic hydrophobic patch density, introducing steric shielding moieties, attenuating affinity for amyloid proteins, and engineering degradable stealth surface layers against corona fouling. Toward this goal, systematic corona profiling could evolve from descriptive characterization to predictive ranking of polymer neurobiological risks.

Mechanistic specificity can also enable targeted biomedical countermeasures. If nanoplastics catalyze amyloid aggregation, inhibitors of small molecules, RNA, metal chelators, molecular chaperones, or engineered nanoparticles may be deployed to disrupt early‐stage protein aggregation via liquid‐liquid phase separation [67, 68, 69] and oligomerization, alleviate nanoplastic‐amyloid protein binding, or drive pathogenic protein aggregation off pathway [70]. Likewise, if NanoEL arises from defined interactions with VE‐cadherin extracellular domains, pharmacological blockade or competitive decoys may prevent nanoplastic paracellular CNS entry, providing a direct causal test of their vascular involvement.

Because nanoplastics can amplify pathology via the gut‐brain axis, often through immune‐activation of the CNS and the enteric nervous system [51, 71], restoring epithelial barrier integrity and microbiome homeostasis with antioxidative and anti‐inflammatory small molecules or nanomedicines represents an additional layer of intervention. Strengthening tight junctions or correcting dysbiosis through supplementation or lifestyle modification may disrupt systemic amplification loops driven by chronic exposure.

From the environmental perspective, polymer chemistries shown to promote pathogenic corona formation or endothelial leakiness should be deprioritized in manufacturing. Development of smart degradable polymers and advanced wastewater nanofiltration technologies could further reduce the environmental burden and human exposure to nanoplastics. Collectively, these strategies establish a translational feedback loop in which mechanistic neurobiology informs materials redesign, therapeutic targeting, and exposure reduction.

### 4.6 Collaborative Networks

International consortia should implement standardized reporting frameworks, preregister study designs, and replicate key findings to improve reproducibility and comparability across laboratories. Owing to the interdisciplinary nature of this research frontier, collaboration across biomedicine, biophysics, chemistry, environmental science, neuroscience, epidemiology, public health, and machine learning is crucial. Such coordinated initiatives can combine expertise, standardize experimental and analytical methodologies, and expedite the translation of mechanistic findings into population‐level insights. By fostering shared resources, aligned methodologies, and cross‐disciplinary communication, collaborative networks will be essential for addressing the challenges of elucidating brain exposure to nanoplastics.

## 5 Outlook

Nanoplastics are everywhere—from the air we breathe to the brain that governs our cognitive function. The convergence of neuroscience, chemistry, and environmental science has revealed a provocative yet realistic possibility that nanoplastics, an unintended byproduct of modern life, may be an unrecognized driver of neurodegeneration entailing a range of neurological and metabolic disorders. Mechanistic data confirm that nanoplastics can accelerate protein aggregation, impair amyloid clearance, propagate pathology systemically, and disrupt endothelial integrity. Recent evidence at environmentally relevant doses strengthens the case for a causal link. Yet causation remains unsubstantiated as highlighted in this article, and the potential links between nanoplastic‐induced neurological disorders and cardiovascular, hepatic, gastrointestinal, and pulmonary diseases remain largely unknown.

Definitive progress in the field requires realistic exposure, methodological improvement, and integration across disciplines, from materials science to epidemiology. If nanoplastics are ultimately established as causal or contributory risk modifiers for neurodegeneration, even modest increases in risk could translate to a substantial population and public healthcare burden, given the ubiquity of exposure and the decades‐long latency of dementia. The implications extend well beyond neuroscience to safer‐by‐design polymer development, exposure surveillance and regulation, and materials policy. They further warrant a precautionary yet evidence‐driven agenda that prioritizes reducing internal nanoplastic exposure while rigorously elucidating their mechanistic and epidemiological contributions. Accordingly, this emerging field represents both a major scientific challenge and a significant societal and public concern.

## Author Contributions

P.C.K. conceived the idea. P.C.K., Y.L., and D.T.L. wrote the manuscript. Y.W. and X.L. surveyed the literature and compiled the reference list. M.X. contributed to the discussion and revision. Y.L. prepared the illustrations.

## Conflicts of Interest

The authors declare no conflict of interest.

## Data Availability

Data sharing not applicable to this article as no datasets were generated or analysed during the current study.
